# Design principles for electrically driven Luttinger liquid-fed plasmonic nanoantennas

**DOI:** 10.1515/nanoph-2022-0782

**Published:** 2023-02-16

**Authors:** Eun Su Jeon, YoonYeong Ko, SeokJae Yoo

**Affiliations:** Department of Physics, Inha University, Incheon, Republic of Korea

**Keywords:** carbon nanotubes, light sources, Luttinger liquid, nanoantenna, one-dimensional materials, tunneling

## Abstract

Electrons injected into one-dimensional (1D) metals are efficiently converted into infrared plasmons because the unique property of the Luttinger liquid, a strongly correlated electronic matter in one-dimensional (1D) metals, prohibits excitations of other quasiparticles. Using the Luttinger liquid behavior, the electrically driven 1D metals can be used as a feed for optical nanoantennas. Nanoantennas can couple the 1D Luttinger liquid plasmons in the feed to the radiating photons in free space. In this work, we suggest design principles for the 1D metallic Luttinger liquid feed and the nanoantennas to obtain high injection and radiation efficiencies, respectively. We expect that our work can promote experimental efforts to realize electrically driven Luttinger liquid-fed nanoantennas and efficient infrared light sources.

## 1 Introduction

Current research for a plasmonic nanoantenna, an optical analog of a radio antenna, aims at a bidirectional operation of antennas; conversion of photons into electrons (*i.e.* a receiver) and its reverse conversion (*i.e.* a transmitter). While the receiving operation can be achieved straightforwardly using semiconductor photodetectors, the transmitting process requires an efficient scheme to drive plasmonic nanoantenna electrically. One of the promising antenna driving schemes is photon emission in the inelastic tunneling process through plasmonic metal nanogaps [1–8]. Some of the electrons tunnel inelastically through the electrically biased nanogap, while most of them elastically tunnel and are transported as the direct current (DC). The inelastic tunneling results in a temporal fluctuation of an electrical current, which is called the quantum shot noise current [2, 3, 5, 8], [9], [10]. The temporal current fluctuations provide photon emission at extremely low efficiency (one photon per ∼10^5^ tunnel electrons) [2, 11] although resonant plasmonic antenna can provide additional enhancement of the photon emission efficiency. Another driving scheme is to use a light-emitting semiconductor feed [12, 13]. Despite its success in visible and near-infrared frequencies, narrow-gap semiconductors become inefficient in mid-infrared (MIR) band emissions because the nonradiative Auger process is dominant [14–16].

Recently, we have proposed that the Luttinger liquid behavior of electrons confined in one-dimensional (1D) paramagnetic metals can provide efficient infrared photon emission when electrons are injected elastically into the 1D metals [17]. In 1D metals, electron–electron interaction becomes dominant, and electrons behave as a strongly correlated electronic liquid, rather than quasi-free particles. Therefore, 1D electronic behavior becomes completely different from higher dimensional metals. The elementary excitation of the Luttinger liquid is plasmons and spinons, which are collective oscillations of charges and spins, respectively. Therefore, in the Luttinger liquid, other excitations, *e.g.* phonons and free electrons, are fundamentally forbidden. This extraordinary property results in the highly efficient injection of electrons into the Luttinger liquid. It has also been proposed that the plasmonic nanoantenna, *e.g.* gold nanorods, nearby can convert the electrically excited Luttinger liquid plasmons into the far-field radiation. The sequential processes of the efficient plasmon excitation and its coupling to the far field can provide a novel route toward electrically driven nanoantennas and efficient infrared lighting.

In this work, we present design principles for plasmonic nanoantennas with the electrically driven Luttinger liquid feed. First of all, we show that electron tunneling through a semiconductor contact can significantly enhance the electron injection efficiency of the Luttinger liquid plasmon. Secondly, we provide design principles for nanoantenna to enhance the Luttinger liquid plasmon-to-radiating photon conversion efficiency. We also suggest a grating nanoantenna design for the practical realization of infrared lighting. We expect our design principles can promote experimental efforts to realize the electrically driven Luttinger liquid-fed plasmonic nanoantennas.

## 2 Results

**Figure 1:**
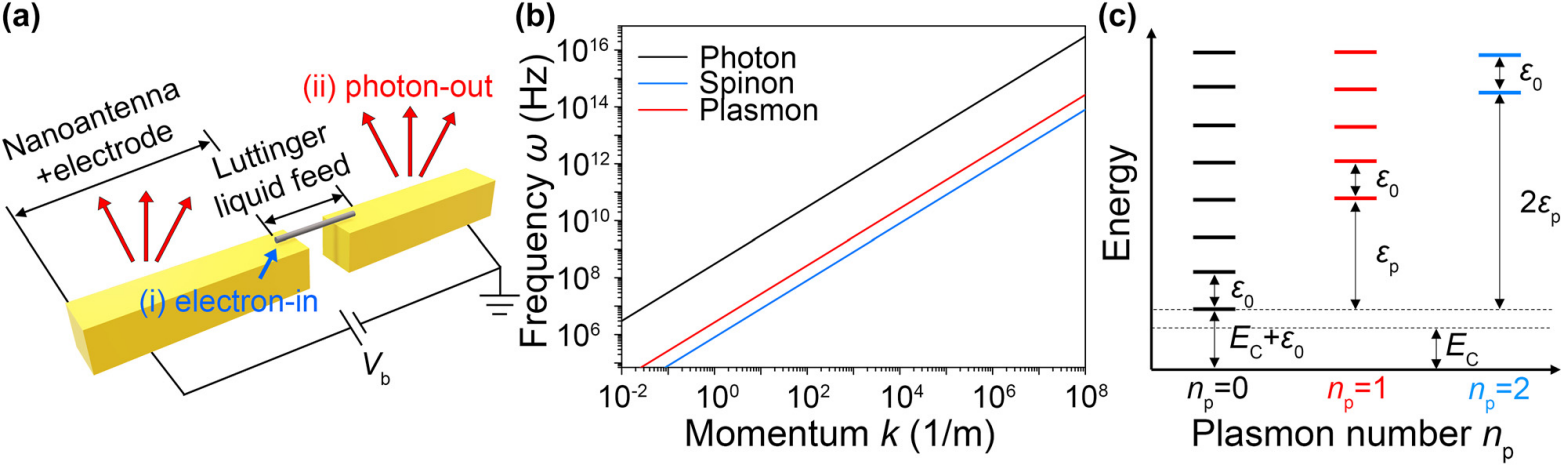
The Luttinger liquid characteristics. (a) Working principle of the electrically driven Luttinger liquid-fed nanoantenna (each step is summarized by blue and red color). The 1D metal at a gap between two gold nanoantennas works as the Luttinger liquid feed when electrons are injected from the gold nanoantenna with a bias voltage *V*_*b*_. (b) The linear dispersion of the Luttinger liquid plasmons (red line: *ω* = *v*_*p*_ *k* = (*v*_*F*_/*g*)*k*), the non-interacting fermi electrons (blue line: *ω* = *v*_*F*_ *k*), and the radiating photons (black line: *ω* = *ck*) with the frequency *ω*, the speed of light *c*, the plasmon velocity *v*_*p*_, the fermi velocity *v*_*F*_, the momentum *k*, the Luttinger liquid interaction parameter *g*, and the speed of light *c*. The dispersion is plotted in a log-log scale with typical parameters of metallic single-walled carbon nanotubes: *g* = 0.3 and *v*_*F*_ = 8 × 10^5^ m/s. (c) The energy spectrum of the Luttinger liquid quantum states is composed of plasmons and spinons. Quantum numbers *n*_*p*_ and *n*_0_ define the energy levels of the plasmon and spinon states (*E*_*C*_: the Coulomb blockade energy, *ε*_0_: the spinon energy, and *ε*_*p*_ = *ε*_0_/*g*: the plasmon energy).

The transmitting operation of the Luttinger liquid-fed nanoantenna follows two steps (Figure 1a): (i) electrons are injected into 1D metals, and they efficiently excite the Luttinger liquid plasmons and spinons. (ii) Nanoantenna helps the electrically excited Luttinger liquid plasmons to couple with the radiating photons. Since the plasmon momentum (red line in Figure 1b) is much larger than the photon momentum (black line in Figure 1c) for a given frequency *ω*, the nanoantenna coupling is needed to radiate the photon in the far field. In this work, we provide design principles for the Luttinger liquid feed and the nanoantenna to enhance the injection efficiency *η*_inj_ (step [i]) and the radiation efficiency *η*_rad_ (step [ii]). In this work, we choose metallic single-walled carbon nanotubes (SWNTs) as the 1D metallic system that hosts the Luttinger liquid. Unique features of the Luttinger liquid have been demonstrated in many experiments including transport [18–20], infrared near-field microscopy [20–22], and electron tunneling-plasmon correlation experiment [20]. The linear dispersion (*e.g.* Figure 1b), the requirement of the paramagnetic 1D metal to host the Luttinger liquid [23], is also robust up to the near-infrared region (∼1 eV) [24, 25]. Although we choose SWNTs as the Luttinger liquid feed material in this work, our antenna design principle does not restrict to SWNTs. Our approach can be applied to any 1D metals, *e.g.* gold atom chains, conducting polymers [26], the mirror-twin boundary of 2D TMDs [27], and twisted WTe_2_ [28]. The Luttinger liquid interaction parameter *g* = *v*_*F*_/*v*_*p*_, the modification of the plasmon velocity *v*_*p*_ compared to the Fermi velocity *v*_*F*_ by the strong electron–electron interaction in 1D metals, determines the electrical and optical behaviors of the Luttinger liquid; *g* < 1, *g* = 1, *g* > 1 correspond to repulsive, vanishing, and attractive interactions, respectively [29]. For example, metallic single-walled carbon nanotubes (SWNT) have *g* ∼ 0.3.

### 2.1 Tailoring electron injection efficiency

Once electrons are injected into the Luttinger liquid, all the injected electrons are converted into plasmons and spinons because they are the elementary excitation. Injected electrons cannot remain as quasi-free electrons in direct current (DC) because quasiparticles are forbidden except for the plasmons and spinons in the Luttinger liquid. Other quasiparticle excitations, which are nonradiative loss channels, are fundamentally forbidden in the Luttinger liquid. This elementary excitation property of the Luttinger liquid is a key to efficient infrared photon emission; the injected electrons get a high chance of conversion into plasmons that can couple to radiating photons. In the Luttinger liquid, there are many quantum states of plasmon and spinon excitations that are defined by plasmon quantum number *n*_*p*_ and spinon quantum number *n*_0_ (*n*_*p*_, *n*_0_ ≥ 0) as depicted in Figure 1c. Among the quantum states, the dipolar plasmon states (*n*_*p*_ = 1) alone are radiative, while the other plasmon states (*n*_*p*_ ≠ 1) and spinon excitations are nonradiative. Therefore, we define the injection efficiency $\eta_{\text{inj}}\left(\varepsilon \right)$ at the energy ε as a ratio of dipolar plasmon excitation power $P_{\text{dip}}\left(\varepsilon \right)$ to total excitation power $P\left(\varepsilon \right)$ , *i.e.* $\eta_{\text{inj}}\left(\varepsilon \right)\equiv P_{\text{dip}}\left(\varepsilon \right)/P\left(\varepsilon \right)$ . In this section, we investigate the electron injection geometry for the Luttinger liquid feed to enhance *η*_inj_.

First of all, the excitation strength of the dipolar plasmon depends on the location of the electron injection. Given 1D metal of length *l*_*LL*_, dipolar plasmon strength is proportional to $\left\{g_{+}+g_{-}\cos\left(2\pi x/l_{LL}\right)\right\}$ where *x* = 0 and *l*_*LL*_ denote both ends of the 1D metal with $g^{\left(\pm \right)}=\left(g^{-1}\pm g\right)/16$ [30]. This implies that dipolar plasmon strength is maximized (minimized) when the electrons are injected at the end (middle) of the 1D metal. Therefore, the electrodes should be located at the end of the 1D metal.

The length of the 1D metal *l*_*LL*_ and the Luttinger liquid interaction parameter *g* affect the injection property. However, *l*_*LL*_ has a constraint imposed by the photon emission wavelength $\lambda_{0}$ . We use the dipolar plasmon states (*n*_*p*_ = 1) for the photon emission. The dipolar plasmons correspond to the lowest Fabry–Perot resonance along the 1D metal, requiring $l_{LL}∼\lambda_{p}/2$ where $\lambda_{p}$ is the Luttinger liquid plasmon wavelength. Note that the reflection phase shift effect at the end of the 1D metal can perturb the half-wavelength condition slightly [17, 31]. If the 1D metal length is away from $l_{LL}∼\lambda_{p}/2$ , the nonradiative higher order Fabry–Perot mode (*i.e.* quadrupolar plasmons) strongly suppresses the photon radiation efficiency *η*_rad_ in step (ii) [*ref: PRL*]. The linear dispersion of the Luttinger liquid plasmon of the momentum $k_{p}=2\pi /\lambda_{p}$ is given by $\omega =\left(v_{F}/g\right)k_{p}$ , where the frequency *ω* is also related to the wavelength of light in free space, $\lambda_{0}=2\pi c/\omega$ . The linear dispersion provides the dipolar resonant length condition, $l_{LL}∼\left(v_{F}/cg\right)\left(\lambda_{0}/2\right)$ .

For the resonant length *l*_*LL*_, we investigate the effect of *l*_*LL*_ and *g* on the injection efficiency *η*_inj_. The energies of the spinon and plasmon states are determined by $\varepsilon_{0}=\pi \hbar v_{F}/l_{LL}$ , and the plasmon energy $\varepsilon_{p}=\varepsilon_{0}/g$ , respectively, with the reduced Planck constant ℏ and the Fermi velocity *v*_*F*_ (*e.g. v*_*F*_ = 8 × 10^5^ m/s for metallic SWNTs). The Luttinger liquid theory predicts that the tunneling density of states (TDOS) $A\left(\varepsilon \right)$ can be decomposed into TDOS of each quantum state $A_{n_{p}n_{0}}\left(\varepsilon \right)$ , *i.e.* [30]

(1) $$A\left(\varepsilon \right)=\underset{n_{p},n_{0}\geq 0}{\sum }A_{n_{p}n_{0}}\left(\varepsilon \right)=\underset{n_{p},n_{0}\geq 0}{\sum }C_{n_{p}n_{0}}\delta \left(\varepsilon -\varepsilon_{n_{p}n_{0}}\right),$$

whose peaks appear at the energy $\varepsilon_{n_{p}n_{0}}=n_{p}\varepsilon_{p}+\left(n_{0}+1/2\right)\varepsilon_{0}+E_{C}$ , where $E_{C}=\left(\varepsilon_{p}^{2}-\varepsilon_{0}^{2}\right)/8\varepsilon_{0}=\varepsilon_{0}\left(1-g^{2}\right)/\left(8g^{2}\right)$ is the Coulomb blockade energy. A coefficient $C_{n_{p}n_{0}}/C_{00}=c_{3/4}^{n_{0}}\sum_{0\leq i\leq j\leq n_{p}}c_{2g^{\left(+\right)}}^{n_{p}-1}c_{g^{\left(-\right)}}^{j-i}c_{g^{\left(-\right)}}^{i}$ describes the TDOS strength of each peak with a coefficient $c_{g}^{n}=\Gamma \left(g+n\right)/\Gamma \left(g\right)\Gamma \left(1+n\right)$ and the Euler-gamma function Γ . The plasmon quantum number *n*_*p*_ = 0, 1, and 2 correspond to the ground state, the dipole state, and the quadrupole state, respectively. $A\left(\varepsilon \right)$ is proportional to the tunneling conductance, *i.e.* $\alpha A\left(\varepsilon \right)=edI\left(\varepsilon \right)/d\varepsilon =e^{2}d^{2}P\left(\varepsilon \right)/d\varepsilon^{2}$ , where *α* and *I* denote a constant prefactor and the tunnel current, respectively. Therefore, we can write

(2) $$I\left(\varepsilon \right)=\alpha \underset{n_{p},n_{0}\geq 0}{\sum }I_{n_{p}n_{0}}\left(\varepsilon \right)=\alpha \underset{n_{p},n_{0}\geq 0}{\sum }C_{n_{p}n_{0}}\theta \left(\varepsilon -\varepsilon_{n_{p}n_{0}}\right),$$

(3) $$\begin{array}{ll} P\left(\varepsilon \right) & =\alpha \underset{n_{p},n_{0}\geq 0}{\sum }P_{n_{p}n_{0}}\left(\varepsilon \right)\\ & =\alpha \underset{n_{p},n_{0}\geq 0}{\sum }C_{n_{p}n_{0}}\left(\varepsilon -\varepsilon_{n_{p}n_{0}}\right)\theta \left(\varepsilon -\varepsilon_{n_{p}n_{0}}\right), \end{array}$$

where the Heaviside theta step function *θ* is defined by *θ*(*x* − *a*) = 1 and 0 for *x* > *a* and *x* ≤ *a*, respectively. Since the dipolar plasmons alone contribute to the radiation, we define the power injected into the dipolar plasmons (*n*_*p*_ = 1) excluding spinon excitations in the form, $P_{\text{dip}}=\alpha \sum_{n_{0}\geq 0}\left(\varepsilon_{p}/\varepsilon_{1n_{0}}\right)P_{1n_{0}}\left(\varepsilon \right)$ . Since we have analytical expressions, Eqs. (1)–(3), and $\eta_{\text{inj}}\left(\varepsilon \right)=P_{\text{dip}}\left(\varepsilon \right)/P\left(\varepsilon \right)$ , we can find the condition of the bias energy *ε*_max_ = *eV*_*b*_ to maximize *η*_inj_. The maximum *η*_inj_ appears when $d\eta_{\text{inj}}\left(\varepsilon \right)/d\varepsilon =0$ , and this leads to a simple condition for *ε*_max_,

(4) $$\frac{P\left(\varepsilon_{\max}\right)}{I\left(\varepsilon_{\max}\right)}=\frac{P_{\text{dip}}\left(\varepsilon_{\max}\right)}{I_{\text{dip}}\left(\varepsilon_{\max}\right)},$$

where the normalized dipole plasmon current $I_{\text{dip}}\equiv \alpha \sum_{n_{0}\geq 0}\left(\varepsilon_{p}/\varepsilon_{1n_{0}}\right)I_{1n_{0}}\left(\varepsilon \right)$ .

**Figure 2:**
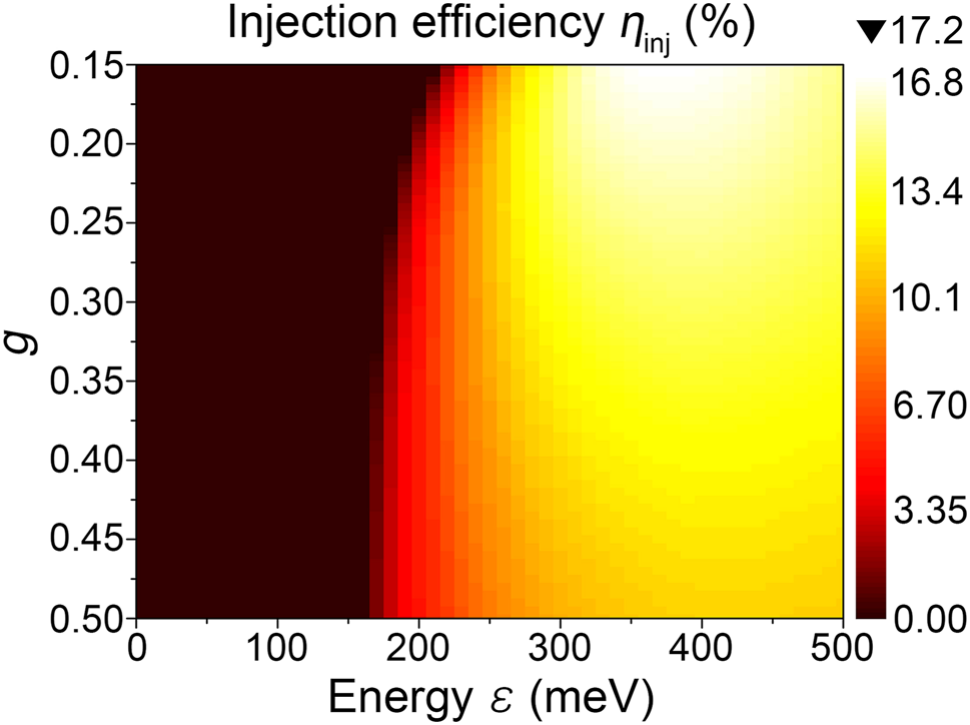
Tailoring the electron injection efficiency *η*_inj_(*ε*) by the Luttinger liquid interaction parameter *g*. The electrons are injected at the end of the 1D metal (*x* = 0 or *l*_*LL*_).

We plot *η*_inj_(*ε*) for varying the Luttinger liquid interaction parameter *g*. Since the dipole plasmon states (*n*_*p*_ = 1) alone contribute to the radiation, *η*_inj_ starts to increase after the lowest energy of the dipole plasmons *ε*_10_ = *ε*_*p*_ + *ε*_0_/2 + *E*_*C*_ = *ε*_0_(3*g* ^2^ + 8*g* + 1)/(8*g* ^2^). Once the electron injection occurs after the Coulomb blockade energy *E*_*C*_ (the boundary between the dark region and the bright region in Figure 2), the injection efficiency maximum *η*_inj_(*ε*_max_) of 17.1% appears at *ε*_max_ = 360 meV for the smallest *g* = 0.15, while *η*_inj_(*ε*_max_) decreases to 11.2% for the largest *g* = 0.5. As *g* decreases, *ε*_max_ becomes larger, while the maximum efficiency *η*_inj_(*ε*_max_) increases. This behavior of better *η*_inj_(*ε*_max_) for small *g* results from two contributions; (i) the dipole plasmon power $P_{1n_{0}}\left(\varepsilon \right)$ is proportional to 1/*g*, *i.e.* $C_{1n_{0}}/C_{00}=c_{3/4}^{n_{0}}/4g$ , according to Eq. (3). (ii) The nonradiative plasmon states start to appear at *ε*_20_ = 2*ε*_*p*_ + *ε*_0_/2 + *E*_*C*_ = *ε*_0_(3*g* ^2^ + 16*g* + 1)/(8*g* ^2^). Therefore, smaller *g* pushes *ε*_20_ to higher energy, *e.g. ε*_20_ = 326 meV and 275 meV for *g* = 0.15 and 0.5, respectively. This implies that we can excite selectively the radiative dipole plasmons (*n*_*p*_ = 1) within the spectral range of interest if *g* is small enough. On the other hand, the 1D metal of larger *g* (*e.g. g* = 0.5) has weaker dipole plasmon power $P_{1n_{0}}\left(\varepsilon \right)\propto 1/g$ , and thus stronger energy is needed to reach *η*_inj_(*ε*_max_) although some higher order nonradiative plasmons are excited.

In addition, the output power of our antenna can be improved by a small *g*. The 1D metal of small *g* also possesses a larger dipole plasmon current, $I_{0}\left(\varepsilon \right)\equiv \underset{n_{0}\geq 0}{\sum }I_{1n_{0}}\left(\varepsilon \right)$ upon the electron injection because the dipole plasmon strength is also proportional to $C_{1n_{0}}/C_{00}=c_{3/4}^{n_{0}}/4g$ according to Eq. (2). Electrically driven dipole plasmon current *I*_0_ plays a role as an optical source in the step (ii), and thus the output power of our antenna scheme scales with (*I*_0_)^2^.

The smallness of *g* provides an additional practical advantage in device fabrication. The resonant 1D metal length *l*_*LL*_ becomes longer for reduced *g* due to the constraint, $l_{LL}∼\left(v_{F}/cg\right)\left(\lambda_{0}/2\right)$ . For example, if we target photon emission at *λ*_0_ = 11 μm, the resonant length of the 1D metal of *g* = 0.5 is given by *l*_*LL*_ = 29.4 nm, which is almost close to the conventional limits of lithography. On the other hand, we can obtain *l*_*LL*_ = 97.8 nm for the 1D metal of *g* = 0.15, making device fabrication easier.

Before concluding this section, we need to mention the experimental way to reduce the Luttinger liquid interaction parameter *g*. *g* varies by the 1D metal species and its dielectric environment. For example, metallic SWNTs follow the logarithmic diameter scaling of *g* [17, 21, 22]; thinner SWNT has smaller *g* (*e.g. g* ∼ 0.29 and 0.36 for diameters of 0.5 nm and 2.0 nm, respectively.) Smaller background permittivity also makes *g* smaller [17, 21]. Recently, extremely small *g* ∼ 0.14 has also been reported in the 1D metal phase in twisted WTe_2_ [28].

It is also noteworthy that other factors can affect the electrical property of the Luttinger liquid feed. First of all, the tunneling current in the 1D metal may experience exponential decrease according to the tunneling barrier characteristics, *e.g.* barrier width and height [32], affecting the resultant injection efficiency. Secondly, we assume the 1D metal feed shorter than 40 nm in our work, but longer feed can suffer from the electron–phonon scattering [33].

To summarize the design principle for high injection efficiency, the following conditions should be met:

(i) Electrons should be injected at the end of the 1D metals rather than the middle because tunneling into the middle suppresses the dipolar plasmons.
(ii) Length of the 1D metals (*l*_*LL*_) is determined by the target wavelength of photon emission ( $\lambda_{0}$ ) and the given Luttinger liquid interaction parameter (*g*), *i.e.* $l_{LL}∼\left(v_{F}/cg\right)\left(\lambda_{0}/2\right)$ .
(iii) Smaller Luttinger liquid interaction parameter *g* leads to higher injection efficiency *η*_inj_ and the dipole current strength *I*_0_.
(iv) The bias energy *eV*_*b*_ = *ε*_max_ to maximize *η*_inj_ can be found near the energy of the lowest quadrupole plasmon (*n*_*p*_ = 2 and *n*_0_ = 0), *ε*_20_ = *ε*_0_(3*g* ^2^ + 16*g* + 1)/(8*g* ^2^) if *g* is small enough.

### 2.2 Tailoring photon radiation efficiency

#### 2.2.1 Calculating the radiation efficiency

To calculate the optical performance of the Luttinger liquid-fed nanoantennas, we include the quantum mechanical properties of the Luttinger liquid in the classical electromagnetic calculation. Resulting from the quantum mechanical electron–electron interaction in 1D metals, the Luttinger liquid has a linear dispersion, $\omega =\left(v_{F}/g\right)k_{p}$ where the frequency *ω* and the Luttinger liquid plasmon momentum *k*_*p*_ are related to the wavelength of light in free space, $\lambda_{0}=2\pi c/\omega$ and the plasmon wavelength $k_{p}=2\pi /\lambda_{p}$ , respectively. Classically, we can determine the permittivity $\varepsilon_{LL}$ of a solid cylinder of radius *R* to follow the linear dispersion of the Luttinger liquid. Given *g*, the permittivity $\varepsilon_{LL}$ can be obtained as a solution to the transcendental equation,

(5) $$\begin{array}{ll} \frac{\varepsilon_{LL}}{\varepsilon_{bg}}\frac{\sqrt{{\tilde{k}}_{p}^{2}-\varepsilon_{bg}k_{0}^{2}}}{\sqrt{{\tilde{k}}_{p}^{2}-\varepsilon_{LL}k_{0}^{2}}} & =-\frac{I_{0}\left(\sqrt{{\tilde{k}}_{p}^{2}-\varepsilon_{LL}k_{0}^{2}}R\right)}{I_{1}\left(\sqrt{{\tilde{k}}_{p}^{2}-\varepsilon_{LL}k_{0}^{2}}R\right)}\\ & \;\times \frac{K_{1}\left(\sqrt{{\tilde{k}}_{p}^{2}-\varepsilon_{bg}k_{0}^{2}}R\right)}{K_{0}\left(\sqrt{{\tilde{k}}_{p}^{2}-\varepsilon_{bg}k_{0}^{2}}R\right)}, \end{array}$$

where the complex plasmon momentum and the free space photon momentum are given by ${\tilde{k}}_{p}=k_{p}\left(1+i/Q\right)=\left(gc/v_{F}\lambda_{0}\right)\left(1+i/Q\right)$ and $k_{0}=2\pi /\lambda_{0}$ , respectively. The imaginary part of the complex momentum is determined by the plasmon quality factor *Q*, which varies by sample preparation quality and environment cleanness. *E*_*bg*_ is the permittivity of the background medium. Since the solid cylinder is thin, we are also able to approximate a solution of Eq. (5) in the simpler form,

(6) $$Re\left\{\varepsilon_{LL}\left(\omega \right)\right\}=-\frac{16}{1-g^{2}}\frac{k_{e}e^{2}}{\varepsilon_{bg}\hbar v_{F}}\frac{v_{F}^{2}}{\pi R^{2}}\frac{1}{\omega^{2}},$$

which works well for a narrow spectral band (*λ*_0_). $k_{e}=1/4\pi \varepsilon_{0}$ is the Coulomb constant, where $\varepsilon_{0}$ is the permittivity of free space. Using Eqs. (5) or (6), we can calculate the optical performance of the Luttinger liquid-fed nanoantennas using numerical electromagnetic solvers, such as the finite-difference time-domain (FDTD) method and the finite element method (FEM). The current source *I*_0_, which corresponds to the electrically driven Luttinger liquid plasmons, is imposed on the 1D metal region. The resultant radiation powers $P_{\text{rad}}{\equiv \oint }_{S}S\cdot ndA$ and Ohmic losses $P_{\Omega ,a\left(LL\right)}\equiv \left(\omega /2\right)Re\int_{V}Im\varepsilon_{a\left(LL\right)}\left(r\right){\left|E\left(r\right)\right|}^{2}dV$ can be classically calculated, where **S**, **n**, **E,** and *V* denote the Poynting vector, the normal vector of the arbitrary closed surface *S*, the electric field, and the lossy material volume, respectively.

To characterize the optical radiation process, *i.e.* the plasmon-to-photon conversion, in step (ii), we need to calculate the radiation efficiency,

(7) $$\begin{array}{ll} \eta_{\text{rad}}=\frac{P_{\text{rad}}}{P_{in}} & =\frac{P_{\text{rad}}}{P_{\text{rad}}+P_{\Omega ,a}+P_{\Omega ,LL}}\\ & =\frac{R_{\text{rad}}}{R_{\text{rad}}+R_{\Omega ,a}+\left({\left|I_{LL}\right|}^{2}/{\left|I_{a}\right|}^{2}\right)R_{\Omega ,LL}}, \end{array}$$

where the total input power *P*_in_ consumed by the whole antenna system is given by a sum of *P*_rad_ and the absorbed powers in the Luttinger liquid feed (*P*_Ω,*LL*_) and in the nanoantenna (*P*_Ω,*a*_). In the last line of Eq. (7), we define the radiation resistance $R_{\text{rad}}\equiv 2P_{\text{rad}}/{\left|I_{a}\right|}^{2}$ , Ohmic resistances $R_{\Omega ,a\left(LL\right)}\equiv \left(l_{a\left(LL\right)}/A_{a\left(LL\right)}\right)Im\left\{1/\omega \varepsilon_{0}\left(1-\varepsilon_{a\left(LL\right)}\right)\right\}$ , and the optical current strengths defined by $\left|I_{a\left(LL\right)}\right|\equiv \left(A_{a\left(LL\right)}/l_{a\left(LL\right)}\right)\int_{V_{a\left(LL\right)}}\left|-i\omega \left(\varepsilon_{a\left(LL\right)}-\varepsilon_{0}\right)E\left(r\right)\right|dr^{3}$ , where $\varepsilon_{a}$ is the permittivity of the nanoantenna. *l*_*a*_, *A*_*a*_, and *V*_*a*_ are the length, cross-section area, and volume of the nanoantenna, while *V*_*LL*_ is the volume of the Luttinger liquid feed. Note that the radiation from the Luttinger liquid feed is negligible because of a huge mismatch between the plasmon wavelength *λ*_*p*_ and the photon wavelength *λ*_0_ (Figure 1b). Eq. (7) shows that a combination of large *R*_rad_, small *R*_Ω_, and large |*I*_*a*_|/|*I*_*LL*_| gives the better radiation efficiency *η*_rad_.

#### 2.2.2 Antenna analysis – the antenna circuit model

**Figure 3:**
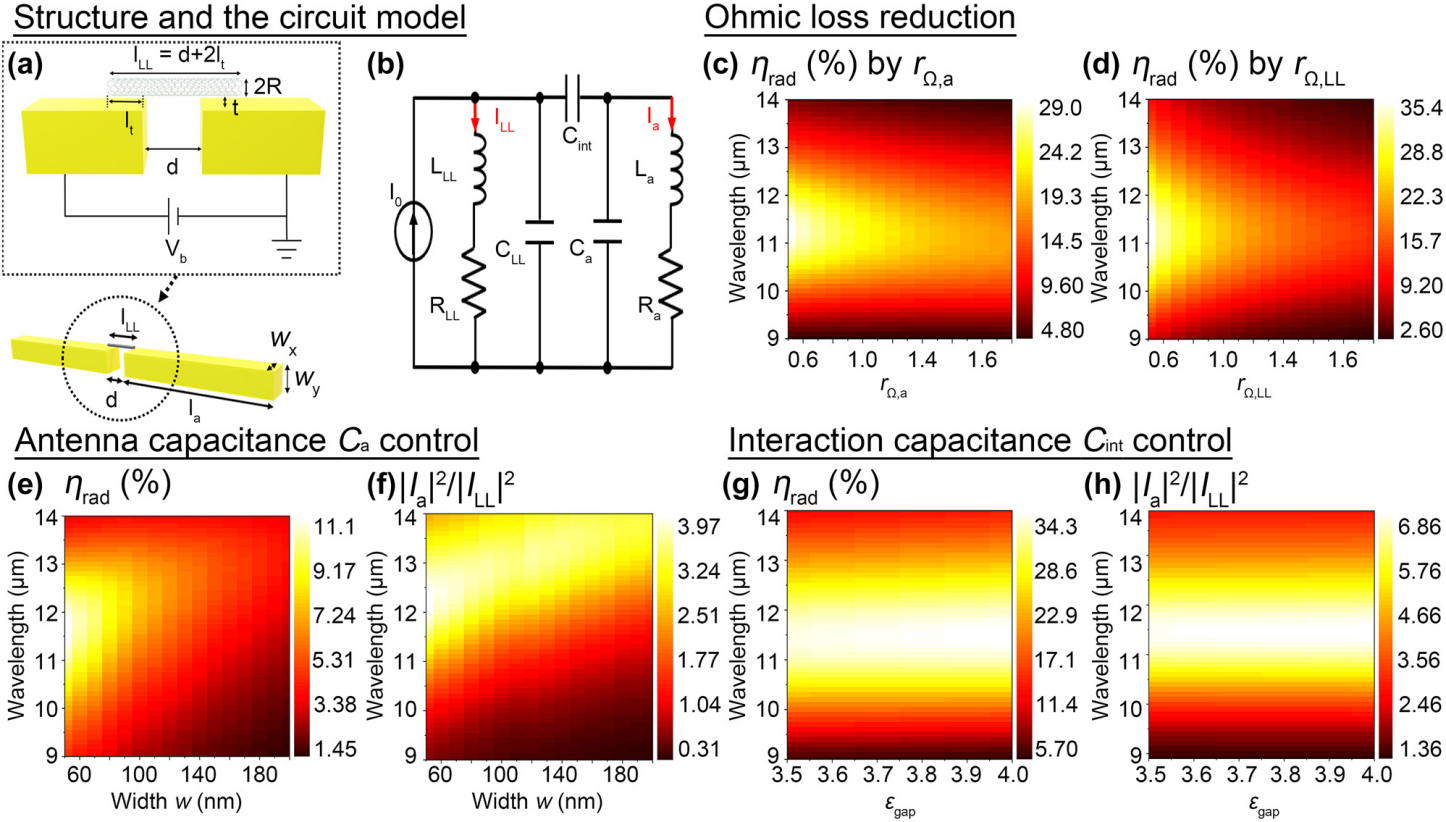
Tailoring the photon radiation efficiency. (a) Schematic drawing of the Luttinger liquid-fed nanoantennas (*w*: the antenna thickness, *d*: the gap distance, *l*_*a*_: the nanoantenna length, *l*_*LL*_ = *d* + 2*l*_*T*_: the Luttinger liquid feed length, *l*_*T*_: the overlap length, *t*: tunneling gap distance). (b) The antenna circuit model with the lumped RLC circuits for the feed and the nanoantenna connected by the interaction capacitor *C*_int_. The whole circuit is driven by the current source *I*_0_ excited by the electron injection. Changes in the radiation efficiency *η*_rad_(*λ*_0_) with the fixed parameters (*l*_*a*_ = 3.8 um, *w* = 60 nm, *l*_*T*_ = 5 nm, *t* = 1 nm, *d* = 30 nm and *ε*_gap_ = 1) by the Ohmic loss reduction in (c) the plasmonic nanoantenna (*r*_Ω,*a*_) and (d) the Luttinger liquid feed (*r*_Ω,*LL*_). The effect of the nanoantenna width *w* on the spectra of (d) the radiation efficiency *η*_rad_(*λ*_0_) and (e) the current strength ratio (|*I*_*a*_(*λ*_0_)|^2^/|*I*_*LL*_(*λ*_0_)|^2^) with the fixed parameters (*l*_*a*_ = 3.8 um, *l*_*T*_ = 5 nm, *t* = 1 nm, and *ε*_gap_ = 1). The effect of the gap permittivity *ε*_gap_ on the spectra of (d) the radiation efficiency *η*_rad_(*λ*_0_) and (e) the current strength ratio (|*I*_*a*_(*λ*_0_)|^2^/|*I*_*LL*_(*λ*_0_)|^2^) with the fixed parameters (*l*_*a*_ = 3.8 um, *w* = 60 nm, *l*_*T*_ = 5 nm, and *t* = 1 nm).

Although Eq. (7) reveals which factors can be improved for better radiation efficiency *η*_rad_, *i.e.* large *R*_rad_, small *R*_Ω_, and large |*I*_*a*_|/|*I*_*LL*_|, it is not straightforward to predict the current strength ratio |*I*_*a*_|/|*I*_*LL*_|. An antenna circuit model in Figure 3b is useful to gain insight to enhance |*I*_*a*_|/|*I*_*LL*_|. We proposed an antenna circuit model composed of two RLC circuits (*i.e.* the 1D metal and the nanoantenna) connected by the interaction capacitor (*C*_int_), and demonstrated that the model can describe the radiation features of the numerical simulations [17]. *R*_*a*(*LL*)_, *L*_*a*(*LL*)_, and *C*_*a*(*LL*)_ are the lumped circuit parameters of the antenna (the Luttinger liquid feed). Note that the lumped resistance *R*_*a*(*LL*)_ includes *R*_rad_ and *R*_Ω_. The circuit model provides the current strength ratio $\left|I_{a}\left(\omega \right)\right|/\left|I_{LL}\left(\omega \right)\right|=\left(L_{LL}/L_{a}\right)\left(\left|Z_{a}\left(\omega \right)\right|/\left|1/i\omega C_{\text{int}}+Z_{a}\left(\omega \right)\right|\right)\sqrt{\left(\omega^{2}+\gamma_{LL}^{2}\right)/\left(\omega^{2}+\gamma_{a}^{2}\right)}$ with the damping constant $\gamma_{a\left(LL\right)}\equiv R_{a\left(LL\right)}/L_{a\left(LL\right)}$ and the antenna impedance *Z*_*a*_. The circuit model prediction for |*I*_*a*_|/|*I*_*LL*_| shows the resonant behavior of the current strength ratio |*I*_*a*_|/|*I*_*LL*_| is governed by *Z*_*a*_(*ω*) resonant at $\omega_{0,a}\equiv \sqrt{1/L_{a}C_{a}}$ , while the optical resonance of the Luttinger liquid feed, *i.e. Z*_*LL*_(*ω*), does not contribute. In the circuit model, the current strength ratio on the antenna resonance *ω* = *ω*_0,*a*_ is given by

(8) $$\frac{{\left|I_{a}\left(\omega_{0,a}\right)\right|}^{2}}{{\left|I_{LL}\left(\omega_{0,a}\right)\right|}^{2}}=\left\{L_{LL}^{2}\left(\omega_{0,a}^{2}+\gamma_{LL}^{2}\right)\right\}\frac{C_{a}}{L_{a}+R_{a}^{2}C_{a}{\left(C_{a}/C_{\text{int}}-1\right)}^{2}}.$$

We can conclude that the antenna circuit model (Eq. (8)) reveals *η*_rad_ ∝ (*R*_*a*_)^−2^, (*L*_*a*_)^−1^, (*C*_*a*_)^−4^, and (*C*_int_)^2^, while the definition of the radiation efficiency (Eq. (7)) shows *η*_rad_ ∝ *R*_rad_, and 1/*R*_Ω_. In the following subsections, we investigate the effect of these contributing parameters on the radiation efficiency *η*_rad_.

#### 2.2.3 Antenna structure and the feed location – the power reciprocity theorem

To obtain high radiation efficiency *η*_rad_, we consider the relationship between the transmitting and receiving operations of the antenna. The transmitting operation is the photon radiation by the antenna for a given current source, and it is of interest in this work. The receiving operation, on the other hand, refers to the photon absorption by the feed for an incoming plane wave. For the latter, there has been numerous researches on near-field enhancement by resonant nanostructures, and they can be good guidance of the antenna structure if we can relate the transmitting and receiving operation. The power reciprocity theorem in electromagnetics relates the absorption cross section $\sigma_{\text{abs}}^{R}$ in the receiving operation with the radiation efficiency *η*_rad_ in the transmitting operation. The superscripts *R* and *T* denote the receiving and transmitting operations, respectively. The absorption cross-section $\sigma_{\text{abs}}^{R}$ is defined by the Ohmic loss of the feed normalized by the intensity of the incoming plane wave of the amplitude $E_{0}^{R}$ , *i.e.* $\sigma_{\text{abs}}^{R}\equiv 2Z_{0}P_{\Omega ,LL}^{R}/{\left|E_{0}^{R}\right|}^{2}$ . *Z*_0_ is the vacuum impedance. The power reciprocity theorem is given by [34]

(9) $$\sigma_{\text{abs}}^{R}=\left\{\frac{\lambda_{0}^{2}}{4\pi }\eta_{L}\eta_{\text{pol}}D^{T}\left({\hat{r}}_{P}\right)\right\}\eta_{\text{rad}},$$

with the load efficiency ( $\eta_{L}\equiv 16P_{in}^{T}P_{\Omega ,LL}^{R}/{\left|\iint_{S_{0}}\left(E^{T}\times H^{R}-E^{R}\times H^{T}\right)\cdot \hat{n}dS\right|}^{2}$ ), the polarization efficiency ( $\eta_{\text{pol}}\equiv {\left|E_{0}^{R}\cdot e^{T}\left({\hat{r}}_{P}\right)\right|}^{2}/\left({\left|E_{0}^{R}\right|}^{2}{\left|e^{T}\right|}^{2}\right)$ ), and the antenna directivity to the measurement position ${\hat{r}}_{p}$ ( $D^{T}\left({\hat{r}}_{P}\right)=4\pi {\left|e^{T}\right|}^{2}/\iint_{\Omega }{\left|e^{T}\right|}^{2}d\Omega$ ). $e^{T}\left({\hat{r}}_{p}\right)$ is the electric field at the far field. Note that $e^{T}\left({\hat{r}}_{p}\right)$ can be numerically calculated from the near-field **E** ^T^(**r**) using the Stratton–Chu equation. Since the power reciprocity theorem, Eq. (9), states that $\sigma_{\text{abs}}^{R}$ and *η*_rad_ are linearly proportional to each other, the Luttinger liquid feed should be located where the near-field enhancement is maximized in the receiving operation.

In the following Sections 2.2.4–2.2.6, we numerically calculate the radiation properties of the rod-type nanoantenna depicted in Figure 3a. The rod-type nanoantenna is the simplest antenna structure, and thus we can obtain general design principles from this example antenna structure. Then, in Section 2.2.7, we expand our discussion to grating nanoantenna that can host multiple Luttinger liquid feeds within the metallic gap.

Our example nanoantenna is composed of two gold rods of the length *l*_*a*_ which have a rectangular cross-section with thickness *w*. Two gold rod nanoantennas have a gap of the distance *d*. The two gold rods host the Luttinger liquid feed of length *l*_*LL*_ = *d* + 2*l*_*T*_ within the gap. The gold rod nanoantennas and the Luttinger liquid feed have an overlap length *l*_*T*_. For the Luttinger liquid feed, we use an SWNT modeled by a solid cylinder of the radius *R* and the Luttinger liquid permittivity *ε*_*LL*_. *ε*_*LL*_ is obtained by a solution of Eq. (5) with *g* = 0.3 and *Q* = 20. The cylindrical 1D Luttinger liquid feed is capacitively connected with the gold rod nanoantennas by the distance *t*. Gold permittivity is taken from the tabulated data [35]. Throughout this work, we do not consider the substrate for sake of simplicity. The current source *I*_0_, which corresponds to the electrically driven dipole plasmon current, is imposed on the SWNT Luttinger liquid feed.

#### 2.2.4 Material quality – Ohmic resistances

Low Ohmic losses can enhance the radiation efficiency *η*_rad_. Ohmic losses occur in both the nanoantenna and the Luttinger liquid feed. To reduce the Ohmic losses in the nanoantenna (Im(*ε*_*a*_)) composed of noble metals such as gold and silver, single crystalline metal can be used. Single crystalline noble metals have ∼80% lower Im(*ε*_*a*_) compared to those of evaporated or template-stripped noble metals in mid-infrared frequencies [35]. Lossless or low-loss dielectric nanoantenna is also able to enhance the radiation efficiency *η*_rad_ if the electrodes are properly prepared for the electron injection to the 1D metal. For the Ohmic loss reduction of the Luttinger liquid feed, high-quality preparation (*e.g.* growth, synthesis, and exfoliation) of the 1D metal are required. The quality factor *Q* determines the imaginary part of the Luttinger liquid permittivity (Im(*ε*_*LL*_)) by the relation *Q* $=\left\{\omega Re\left(d\varepsilon_{LL}/d\omega \right)\right\}/\left\{2Im\left(\varepsilon_{LL}\right)\right\}$ . The quality factor *Q* of 5 ∼ 20 is experimentally achieved in the infrared Luttinger liquid plasmons of SWNTs [20], [21], [22, 36]. Encapsulation with an atomically flat insulator such as hexagonal boron nitride (hBN) also improves the Luttinger liquid plasmon quality factor empirically [20, 22, 36]. The mid-infrared frequency has rich bands of molecular vibrations, possibly quenching the Luttinger liquid plasmons. Therefore, environmental cleanness is also required. In Figure 3c and d, we calculate the effect of Ohmic losses of the nanoantenna and the Luttinger liquid feed on *η*_rad_ by imposing the modified imaginary parts of the permittivity, *r*_Ω,*a*_Im(*ε*_*a*_) and *r*_Ω,*LL*_Im(*ε*_*LL*_), where we define the Ohmic loss reduction coefficient *r*_Ω*a*(*LL*)_ of the nanoantenna (the Luttinger liquid feed). Figure 3c and d shows *η*_rad_ for different *r*_Ω,*a*_ (*r*_Ω,*LL*_) = 0.5, 1.0, 2.0 for *r*_Ω,*LL*_ (*r*_Ω,*a*_) is fixed to the unity. As expected, *η*_rad_ increases by the Ohmic loss reduction; *η*_rad_ is improved from 23.3% to 29.0% by the Ohmic loss reduction of *r*_Ω,*a*_ = 0.5 in the gold nanoantenna. On the other hand, the effect of the Ohmic loss reduction of the SWNT Luttinger liquid feed, *i.e. r*_Ω,*LL*_ = 0.5, is much effective than that in the nanoantenna; *η*_rad_ is improved from 23.3% to 35.4%. This originates from the strong plasmonic field enhancement in the vicinity of the feed. The Luttinger liquid plasmons along the feed provide the plasmonic near-field enhancement, while the strong capacitive gap between the feed and the nanoantenna also provide the additional enhancement. Therefore, the absorption mainly occurs in the feed, making the Ohmic loss reduction in the feed important.

#### 2.2.5 Antenna geometry – antenna impedance

The antenna circuit model suggests that careful tuning of antenna impedances, *i.e.* antenna resistance *R*_*a*_, capacitance *C*_*a*_, and inductance *L*_*a*_, can enhance the radiation efficiency because *η*_rad_ ∝ (*R*_*a*_)^−2^, (*L*_*a*_)^−1^, and (*C*_*a*_)^−4^. In this subsection, we study the effect of these three parameters. First of all, we change the antenna inductance *L*_*a*_ and capacitance *C*_*a*_ by varying the antenna width *w* = *w*_*x*_ = *w*_*y*_. According to the antenna theory [37, 38], a thinner antenna (*i.e.* small *r*_*a*_) has smaller antenna capacitance (*i.e.* smaller *C*_*a*_); for example, a cylindrical wire of length *l*_*a*_ and radius *r*_*a*_ has antenna capacitance $C_{a}=\varepsilon_{bg}l_{a}/\ln\left(l_{a}/r_{a}\right)$ where *l*_*a*_/*r*_*a*_ > 1, while the antenna gap capacitance is simply given by *C*_gap_ = *ε*_0_ *A*_*a*_/*d*. On the other hand, the peak wavelength of (|*I*_*a*_|/|*I*_*LL*_|)^2^ in Figure 3f remains almost the same according to varying *w*. The constant resonance frequency $\omega_{0,a}\equiv \sqrt{1/L_{a}C_{a}}$ for decreasing *w* implies that the antenna impedance *L*_*a*_ increases as the same rate of decrease in the antenna capacitance *C*_*a*_. However, the circuit model prediction, Eq. (8), provides *η*_rad_ ∝ (*L*_*a*_)^−1^, and (*C*_*a*_)^−4^, making the effect of the antenna capacitance *C*_*a*_ dominate *η*_rad_. In Figure 3e, a numerical result shows that *η*_rad_ increases when the width *w* decreases as expected by the antenna theory (Please find Figure S1 in the Supplementary Materials for additional data including a bowtie antenna).

The bandwidth of *η*_rad_(*λ*_0_) in Figure 3e also broadens for small *w*, while that of (|*I*_*a*_|/|*I*_*LL*_|)^2^ peak remains almost the same. This behavior can be understood as an increase in the Ohmic resistance $R_{\Omega ,a}\equiv \left(l_{a}/A_{a}\right)Im\left\{1/\omega \varepsilon_{0}\left(1-\varepsilon_{a}\right)\right\}$ due to the smaller cross-section area *A*_*a*_ = *w* ^2^. Note that a combination of *w*_*x*_ and *w*_*y*_ with the same cross-section area *A*_*a*_ = *w*_*x*_ *w*_*y*_ gives the same results in *η*_rad_ and (|*I*_*a*_|/|*I*_*LL*_|)^2^ (Please find Figure S2 in the Supplementary Materials for corresponding plots of *R*_rad_, *R*_Ω,*a*_, and *R*_Ω,*LL*_.)

The antenna resistance *R*_*a*_ results from the Ohmic resistances (*R*_Ω,*a*_ and *R*_Ω,*LL*_) and the radiation resistance (*R*_rad_). We have already seen in Section 2.2.4, smaller Ohmic resistances *R*_Ω,*a*_ and *R*_Ω,*LL*_ can improve *η*_rad_. The radiation resistance cannot be controlled once the antenna structure is determined. For example, the rod-type antenna has the radiation resistance is given $R_{\text{rad}}=\left(2\pi /3\right)n_{bg}Z_{0}{\left(l_{a}/\lambda_{0}\right)}^{2}$ where *Z*_0_, *l*_*a*_, and *n*_*bg*_ are the vacuum impedance, the antenna length, and the refractive index of the background medium, respectively [37]. Since our antenna is resonant, *l*_*a*_ is restricted to ∼*λ*_0_/2, providing a constant $R_{\text{rad}}=\left(\pi /6\right)n_{bg}Z_{0}$ . Therefore, future research for antenna structures with *R*_rad_ higher than rod-type antennas is required.

#### 2.2.6 Capacitive feed-antenna coupling – interaction capacitance

The Luttinger liquid feed and the gold nanoantenna are connected capacitively (Figure 3a and b). We hypothesize that the interaction capacitance *C*_int_ is proportional to the area of the capacitive interface *A* and the gap permittivity *ε*_gap_, while it is inversely proportional to the feed-antenna distance *t*, as a parallel plate capacitor does. The easiest way to obtain high gap permittivity is van der Waals insulators such as hBN, which are used as an atomically flat substrate for van der Waals materials and SWNTs [20, 22, 36]. The effective permittivity of hBN also reaches ∼3.7 in the mid-infrared frequencies [39, 40] Figure 3g and h shows the radiation efficiency *η*_rad_ and the current strength ratio (|*I*_*a*_|/|*I*_*LL*_|)^2^ according to the permittivity of the gap between SWNT and gold nanorods. With an air gap (*ε*_gap_ = 1), the radiation efficiency *η*_rad_ is achieved by 17%. As *ε*_gap_ increases up to 3.7, *η*_rad_ doubles, *i.e.* ∼34%. (|*I*_*a*_|/|*I*_*LL*_|)^2^ in Figure 3h also shows a stronger optical current flowing through the nanoantenna for larger *ε*_gap_, while the Luttinger liquid feed loses more optical currents. As we have seen in Figure 3g and h, an increase in the gap permittivity is highly effective to increase *η*_rad_. We expect further enhancement of *η*_rad_ if we fill the gap with high-k dielectrics [41, 42] and phononic materials that the reststrahlen band increases the gap permittivity in infrared frequencies [43]. Note that we demonstrated that an increase in the overlap length, *i.e.* larger capacitive area, is also able to improve *η*_rad_ in our previous work [17].

#### 2.2.7 Array of the Luttinger liquid feed

**Figure 4:**
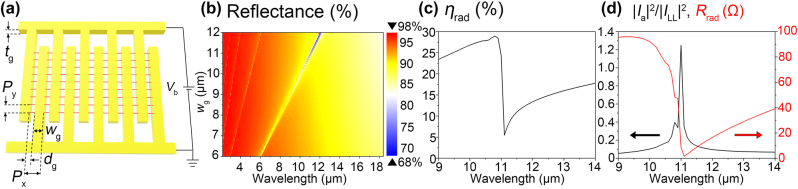
Grating-based nanoantenna fed by the Luttinger liquid array. (a) A device structure (*w*_*g*_: grating width, *t*_*g*_: grating thickness, *d*_*g*_: gap distance, *l*_*LL*_ = *d* + 2*l*_*T*_: the feed length, *l*_*T*_: the overlap length, *P*_*x*_ = *w*_*g*_ + *d*_*g*_ and *P*_*y*_: the period along the *x* and *y*-direction, respectively). Spectrum of (b) the reflectance according to the grating width *w*_*g*_ upon the normally incident plane wave in the receiving operation. (c) The radiation efficiency *η*_rad_(*λ*_0_), (d) the current strength ratio (|*I*_*a*_(*λ*_0_)|^2^/|*I*_*LL*_(*λ*_0_)|^2^), and the radiation resistance *R*_rad_(*λ*_0_) for *w*_*g*_ = 11 μm in the transmitting operation by the feed current source *I*_0_.

Integration of the multiple Luttinger liquid feeds to the nanoantenna can promote efforts to realize proof-of-concept experiments and practical light sources. Figure 4a shows a grating-type nanoantenna fed by an array of the 1D metals hosting the Luttinger liquid. To bias the Luttinger liquid feed array at the middle, upper and bottom electrodes are connected to the grating nanoantenna, forming a structure similar to two crossed combs. The 1D metal array can be obtained experimentally in twisted WTe_2_ [28], while it is also expected to be found in bilayer graphene by theoretical prediction [44, 45]. In Figure 4, grating width *w*_*g*_ varies while *d*_*g*_ is fixed to 30 nm, *i.e. l*_*LL*_ = 40 nm with *l*_*T*_ = 5 nm. Grating thickness is also fixed to *t*_*g*_ = 60 nm. The period along the *y*-direction is assumed to be *P*_*y*_ = 1 um for simplicity to avoid heavy numerical costs. The period along the *x*-direction is given by *P*_*x*_ = *w*_*g*_ + *d*_*g*_.

As we have studied in rod-type nanoantennas, the radiation efficiency is governed by the antenna resonance. To obtain the antenna resonance in the spectral region of interest, we first calculate the reflectance of the grating for the normally incident plane wave in the receiving operation. Figure 4b shows the grating resonances appearing when *w*_*g*_ ∼ *λ*_0_ with a sharp Fano lineshape, an asymmetric spectrum composed of subsequent peak and dip. In transmitting operation by the feed current source *I*_0_, Figure 4c and d plot the radiation efficiency *η*_rad_(*λ*_0_) (Figure 4c), the current strength ratio (|*I*_*a*_(*λ*_0_)|^2^/|*I*_*LL*_(*λ*_0_)|^2^) (black line in Figure 4d), and the radiation resistance *R*_rad_(*λ*_0_) (red line in Figure 4d) for *w*_*g*_ = 11 μm. Maximum radiation efficiency *η*_rad_ of ∼30% is achieved at the peak wavelength of *λ*_0_ = 11 μm in Figure 4c. This is higher than that of 23% in the rod-type nanoantenna with the same width of *w* = 60 nm and resonance wavelength of *λ*_0_ = 11 μm in our previous work [17]. The current strength ratio (|*I*_*a*_(*λ*_0_)|^2^/|*I*_*LL*_(*λ*_0_)|^2^) does not show the asymmetric Fano lineshape, while the radiation resistance *R*_rad_(*λ*_0_) does. The peak of the current strength ratio is located at *λ*_0_ = 11 μm the center of the Fano peak and dip in *η*_rad_(*λ*_0_). We numerically demonstrate that the grating-type nanoantennas are also able to provide high radiation efficiency *η*_rad_, promising experimental realization of the electrically driven Luttinger liquid-fed nanoantennas.

## 3 Conclusions

We have proposed design principles to enhance the injection efficiency *η*_inj_ and the radiation efficiency *η*_rad_ of the Luttinger liquid-fed nanoantennas. Since the photon emission in our nanoantenna scheme is mediated by the two steps (*i.e.* electron injection and photon radiation), the resultant quantum efficiency *η*_Q_ is given by *η*_Q_ = *η*_inj_ × *η*_rad_. In our previous work, we obtained *η*_Q_ ∼3% without the optimization using the design principles we present in this work [17]. Here, we suggest that a much higher *η*_Q_ is achievable via tailoring the electron injection scheme and the nanoantenna structure. Also, we propose that the grating nanoantennas can provide radiation efficiency *η*_rad_, even higher than rod nanoantennas. The grating nanoantennas can host multiple Luttinger liquid feeds within the metallic gap, making the photon emission intensity larger. This may be an advantage for proof-of-concept of the electrically driven Luttinger liquid-fed nanoantennas. Before concluding, we also emphasize that our scheme of the Luttinger liquid-fed nanoantennas works only on an electron-in/photon-out operation. However, its reverse process, *i.e.* a photon-in/electron-out operation, may be possible if we can extract electrons from optically excited Luttinger liquid plasmons. Research toward the bidirectional operation of the Luttinger liquid-fed nanoantenna can be an interesting direction to pursue.

## Supplementary Material

Supplementary Material Details

## References

[1] Parzefall M., Bharadwaj P., Jain A., Taniguchi T., Watanabe K., Novotny L. (2015). Antenna-coupled photon emission from hexagonal boron nitride tunnel junctions. Nat. Nanotechnol..

[2] Wang P., Krasavin A. V., Nasir M. E., Dickson W., Zayats A. V. (2018). Reactive tunnel junctions in electrically driven plasmonic nanorod metamaterials. Nat. Nanotechnol..

[3] Zhang C., Hugonin J. P., Coutrot A. L., Sauvan C., Marquier F., Greffet J. J. (2019). Antenna surface plasmon emission by inelastic tunneling. *Nat. Commun.*.

[4] Chen P., Wang W., Lin N., Du S. (2014). Manipulating photon emission efficiency with local electronic states in a tunneling gap. Opt. Express.

[5] Kern J., Kullock R., Prangsma J., Emmerling M., Kamp M., Hecht B. (2015). Electrically driven optical antennas. Nat. Photonics.

[6] Prangsma J. C., Kern J., Knapp A. G. (2012). Electrically connected resonant optical antennas. Nano Lett..

[7] Bharadwaj P., Bouhelier A., Novotny L. (2011). Electrical excitation of surface plasmons. Phys. Rev. Lett..

[8] Parzefall M., Novotny L. (2019). Optical antennas driven by quantum tunneling: a key issues review. Rep. Prog. Phys..

[9] Kaasbjerg K., Nitzan A. (2015). Theory of light emission from quantum noise in plasmonic contacts: above-threshold emission from higher-order electron-plasmon scattering. Phys. Rev. Lett..

[10] Braun K., Laible F., Hauler O. (2018). Active optical antennas driven by inelastic electron tunneling. Nanophotonics.

[11] Bigourdan F., Hugonin J. P., Marquier F., Sauvan C., Greffet J. J. (2016). Nanoantenna for electrical generation of surface plasmon polaritons. Phys. Rev. Lett..

[12] Maksymov I. S., Staude I., Miroshnichenko A. E., Kivshar Y. S. (2012). Optical yagi-uda nanoantennas. Nanophotonics.

[13] Ma L., Yu P., Wang W. (2021). Nanoantenna-enhanced light-emitting diodes: fundamental and recent progress. Laser Photon. Rev..

[14] Aytac Y., Olson B. V., Kim J. K. (2016). Bandgap and temperature dependence of auger recombination in InAs/InAsSb type-II superlattices. J. Appl. Phys..

[15] Hjalmarson H. P., Kurtz S. R. (1996). Electron auger processes in mid-infrared InAsSb/InGaAs heterostructures. Appl. Phys. Lett..

[16] Vinter B. (2002). Auger recombination in narrow-gap semiconductor superlattices. Phys. Rev. B.

[17] Yoo S., Zhao S., Wang F. (2021). Infrared light-emitting devices from antenna-coupled luttinger liquid plasmons in carbon nanotubes. Phys. Rev. Lett..

[18] Bockrath M., Cobden D. H., McEuen P. L. (1997). Single-electron transport in ropes of carbon nanotubes. Science.

[19] Bockrath M., Cobden D. H., Lu J. (1999). Luttinger-liquid behaviour in carbon nanotubes. Nature.

[20] Zhao S., Wang S., Wu F. (2018). Correlation of electron tunneling and plasmon propagation in a Luttinger liquid. Phys. Rev. Lett..

[21] Shi Z., Hong X., Bechtel H. A. (2015). Observation of a Luttinger-liquid plasmon in metallic single-walled carbon nanotubes. Nat. Photonics.

[22] Wang S., Wu F., Zhao S. (2019). Logarithm diameter scaling and carrier density independence of one-dimensional Luttinger liquid plasmon. Nano Lett..

[23] Voit J. (2000). A brief introduction to Luttinger liquids. AIP Conf. Proc..

[24] Ouyang M., Huang J. L., Lieber C. M. (2002). One-dimensional energy dispersion of single-walled carbon nanotubes by resonant electron scattering. Phys. Rev. Lett..

[25] Liu K., Deslippe J., Xiao F. (2012). An atlas of carbon nanotube optical transitions. Nat. Nanotechnol..

[26] Wang J., Niu J., Shao B. (2021). A tied Fermi liquid to Luttinger liquid model for nonlinear transport in conducting polymers. Nat. Commun..

[27] Jolie W., Murray C., Weiß P. S. (2019). Tomonaga-Luttinger liquid in a box: electrons confined within MoS2 mirror-twin boundaries. Phys. Rev. X.

[28] Wang P., Yu G., Kwan Y. H. (2022). One-dimensional Luttinger liquids in a two-dimensional moiré lattice. Nature.

[29] Voit J. (1995). One-dimensional Fermi liquids. Rep. Prog. Phys..

[30] Kane C., Balents L., Fisher M. (1997). Coulomb interactions and mesoscopic effects in carbon nanotubes. Phys. Rev. Lett..

[31] Luo X., Hu C., Lyu B. (2020). Reflection phase shift of one-dimensional plasmon polaritons in carbon nanotubes. Phys. Rev. B.

[32] Simeone F. C., Yoon H. J., Thuo M. M., Barber J. R., Smith B., Whitesides G. M. (2013). Defining the value of injection current and effective electrical contact area for EGaIn-based molecular tunneling junctions. J. Am. Chem. Soc..

[33] Park J.-Y., Rosenblatt S., Yaish Y. (2004). Electron−Phonon scattering in metallic single-walled carbon nanotubes. Nano Lett..

[34] de Hoop A. T., de Jong G. (1974). Power reciprocity in antenna theory. Proc. Inst. Electr. Eng..

[35] Olmon R. L., Slovick B., Johnson T. W. (2012). Optical dielectric function of gold. Phys. Rev. B.

[36] Wang S., Yoo S., Zhao S. (2021). Gate-tunable plasmons in mixed-dimensional van Der Waals heterostructures. Nat. Commun..

[37] Eggleston M. S., Messer K., Zhang L., Yablonovitch E., Wu M. C. (2015). Optical antenna enhanced spontaneous emission. Proc. Natl. Acad. Sci..

[38] Staffaroni M., Conway J., Vedantam S., Tang J., Yablonovitch E. (2012). Circuit analysis in metal-optics. Photonics Nanostructures – Fundam. Appl..

[39] Kumar A., Low T., Fung K. H., Avouris P., Fang N. X. (2015). Tunable light–matter interaction and the role of hyperbolicity in graphene–HBN system. Nano Lett..

[40] Woessner A., Lundeberg M. B., Gao Y. (2015). Highly confined low-loss plasmons in graphene–boron nitride heterostructures. Nat. Mater..

[41] Yang A. J., Han K., Huang K. (2022). Van der Waals integration of high-κ perovskite oxides and two-dimensional semiconductors. Nat. Electron..

[42] Wang J., Lai H., Huang X. (2022). High-κ van Der Waals oxide MoO3 as efficient gate dielectric for MoS2 field-effect transistors. Materials.

[43] Foteinopoulou S., Devarapu G. C. R., Subramania G. S., Krishna S., Wasserman D. (2019). Phonon-polaritonics: enabling powerful capabilities for infrared photonics. Nanophotonics.

[44] Killi M., Wei T. C., Affleck I., Paramekanti A. (2010). Tunable Luttinger liquid physics in biased bilayer graphene. Phys. Rev. Lett..

[45] Chen C., Neto A. H. C., Pereira V. M. (2020). Correlated states of a triangular net of coupled quantum wires: implications for the phase diagram of marginally twisted bilayer graphene. Phys. Rev. B.

