# Supplementary material for: Design principles for electrically driven Luttinger liquid-fed plasmonic nanoantennas
Source: Nanophotonics. 2023 Feb 16;12(13):2507–16. doi: 10.1515/nanoph-2022-0782 (PMC11501488; doi:10.1515/nanoph-2022-0782)
Supplement: Supplementary file 1 — Supplementary Material Details [file j_nanoph-2022-0782_suppl.pdf]

Eun Su Jeon#, YoonYeong Ko#, and SeokJae Yoo*

Supplementary Materials: Design Principles for Electrically Driven Luttinger Liquid-fed Plasmonic Nanoantennas

1 Additional data for other antenna structures

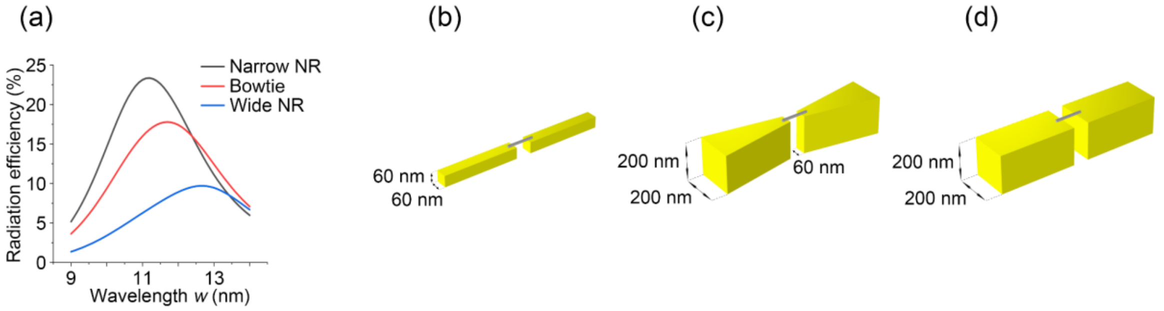

Figure S1. Comparison of three different antennas. (a) Radiation efficiencies of (b) a narrow rod, (c) a bowtie, and (d) a wide rod antenna. Antenna widths are described in (b)-(d).

2 Contributing factors of the radiation efficiency

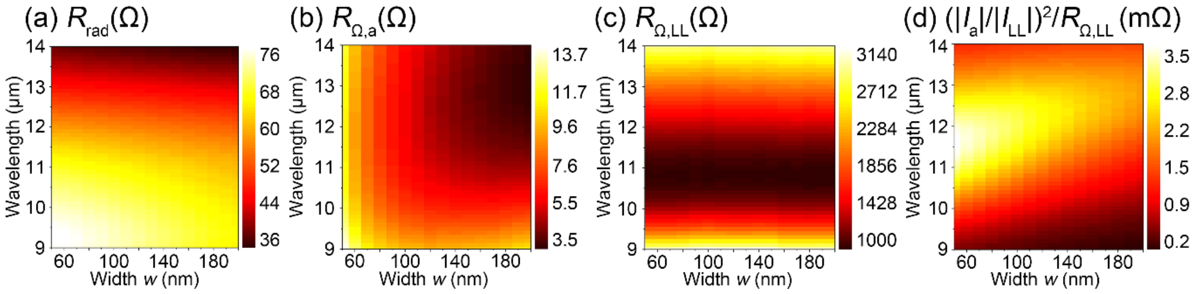

Figure S2. Components of the radiation efficiency $\eta_{rad}(\lambda_0)$; (a) Radiation resistance of the whole system, (b) Ohmic resistance of the antenna, and (c) Ohmic resistance of the Luttinger liquid feed in terms of the antenna width w.

* **Corresponding author: SeokJae Yoo**, Department of Physics, Inha University, Incheon, Republic of Korea, E-mail: seokjaeyoo@inha.ac.kr. https://orcid.org/0000-0002-6438-7123.
Eun Su Jeon and YoonYeong Ko, Department of Physics, Inha University, Incheon, Republic of Korea. https://orcid.org/0000-0001-5135-9353 (Eun Su Jeon), https://orcid.org/0000-0003-0667-7656 (YoonYeong Ko).
# These authors contributed equally to this work.
